# Head Position During Sleep: Potential Implications for Patients with Neurodegenerative Disease

**DOI:** 10.3233/JAD-180697

**Published:** 2019-01-22

**Authors:** Daniel J. Levendowski, Charlene Gamaldo, Erik K. St. Louis, Luigi Ferini-Strambi, Joanne M. Hamilton, David Salat, Philip R. Westbrook, Chris Berka

**Affiliations:** a Advanced Brain Monitoring, Carlsbad, CA, USA; b Department of Neurology, Johns Hopkins University School of Medicine, Baltimore, MD, USA; c Center for Sleep Medicine, Departments of Neurology and Medicine, Mayo Clinic College of Medicine and Science, Rochester, MN, USA; d Department of Clinical Neurosciences, San Raffaele Scientific Institute, Sleep Disorders Center Università Vita-Salute San Raffaele, Milan, Italy; e Advanced Neurobehavioral Health, San Diego, CA, USA; f Harvard Medical School, Boston, MA, USA; g Massachusetts General Hospital, Boston, MA, USA

**Keywords:** Head position, neurodegeneration, obstructive sleep apnea, sleep, supine

## Abstract

**Background:**

The characterization of sleep in those with neurodegenerative disease (NDD) is essential in understanding the potential neurobiological mechanisms that underlie the connection between sleep disruption and NDD manifestations and progression.

**Objective:**

Explore the inter-relationships between NDD and age, sex, diagnosis of obstructive sleep apnea, snoring, and duration of sleep time with the head in the supine and non-supine positions.

**Methods:**

A case-control design was used to evaluate differences in sleep position obtained from multi-night, in-home Sleep Profiler recordings in 45 patients with diagnosed NDD (24 with mild cognitive impairment, 15 with Alzheimer’s disease, and 6 with Lewy Body, Parkinson’s, or other dementias) and 120 age-sex matched controls with normal cognition (NC).

**Results:**

The frequency of supine sleep >2 h/night was significantly greater in the NDD than in the NC group (*p* < 0.001, odds ratio = 3.7), and remained significant after controlling for age, sex, snoring, and obstructive sleep apnea diagnosis (*p* = 0.01). There were no group differences in nocturnal mobility i.e., number of head position changes/h.

**Conclusion:**

This study demonstrates the utility of in-home measurements of sleep in defining the association of supine sleep position with neurodegenerative disorders. Our findings warrant further investigation, particularly in light of the recent evidence suggesting that sleep may an active role in the brain’s ability to clear CNS neurotoxins and metabolites.

## INTRODUCTION

Sleep abnormalities are highly prevalent in patients with neurodegenerative disease (NDD), often appearing in the pre-clinical stage long before cognitive decline or other objective neurological deficits are detected. The association between sleep disturbances and neurodegeneration may be bidirectional, as sleep disturbances may alternatively cause or result from neurodegenerative processes in the brain [1]. The presence of clinical sleep disorders has been linked with increased risk of future NDD, for instance, a recent study found patients with primary insomnia as young adults had a higher risk of developing dementia than those without primary insomnia [2]. Late-midlife obstructive sleep apnea (OSA) and short sleep duration has been linked to the manifestation of dementia in later life [3].

One mechanism suggested as underlying the relationship between sleep and NDD is the glymphatic system which clears soluble amyloid-β (Aβ) and likely other neurotoxic proteins from the brain, and which is selectively active during sleep [4–6]. Decreased sleep duration and disruption in nightly sleep have been shown to be associated with inefficient Aβ clearance, implying that sleep disturbance could lead to Aβ and other toxic proteins accumulating in the brain, potentially leading to an increased risk for neurodegeneration [7–9]. Even a single night of sleep deprivation was recently shown to lead to increased brain Aβ production, and acute sleep deprivation was also very recently shown to increase Aβ accumulation in the right hippocampus and thalamus, correlating with negative mood [7, 8, 10–13]. Gravity also affects the movement and distribution of blood out of the brain, and therefore characteristic sleep positions may also play a role in the efficiency of protein clearance from the brain [14–19].

The aim of this study was to conduct an exploratory investigation into the potential relationship between characteristic sleep patterns in a community dwelling NDD cohort in comparison to age-sex matched controls with normal cognitive function.

## MATERIALS AND METHODS

### Subjects

Sleep records selected for this study were acquired from a comprehensively characterized NDD cohort participating in three IRB-approved, multi-site, longitudinal studies. NDD records were included if the sleep time exceeded 4 h with less than 10% of recording time rejected due to artifact. All but two of the studies were two-night recordings. The NDD cohort used for this study included 24 subjects diagnosed with mild cognitive impairment (MCI), 15 with Alzheimer’s disease (AD), 3 with Parkinson’s disease (PD), 2 with dementia with Lewy bodies (DLB), and 1 with unspecified dementia (Other). All had either a self-reported memory complaint or noticeable memory impairment reported by a family member/caregiver.

All of the AD, PD, and DLB and 14 of the MCI participants were recruited from the greater San Diego area. Patients were either enrolled in the Shiley-Marcos University of California, San Diego (UCSD) Alzheimer’s Disease Research Center (ADRC) longitudinal project and agreed to be contacted for additional study, or were recruited from community neurologists. Eligible participants with AD were diagnosed with dementia by board-certified neurologists with expertise in dementia and movement disorders based on criteria specified in the Diagnostic and Statistical Manual of Mental Disorders (DSM-5). Briefly, diagnostic criteria for AD were: a) presence of objective cognitive impairment (≥1.5 standard deviations) in the memory domain plus at least one other cognitive domain, b) decline in activities of daily living due to cognitive impairment, and c) absence of other medical or mental disease that explained the syndrome. Diagnostic criteria for MCI were: a) presence of objective cognitive impairment (≥1.5 standard deviation (SD)) in the memory domain, b) absence of decline in activities of daily living, and c) absence of medical or mental disease that explained the syndrome. Patients with probable DLB were diagnosed based on McKeith criteria [20]. Participants with “other dementia” distinction were individuals who met DSM-5 criteria for major neurocognitive disorder, but in whom a probable etiology was not clear (i.e., deficits were not typical of AD or DLB).

Ten MCI patients enrolled in this study through the Brain Aging and Dementia Laboratory at Massachusetts General Hospital and were referred through the Massachusetts General Hospital Alzheimer’s Disease Research Center or participated in a local longitudinal cohort. The MCI designation was assigned to non-demented participants with Mini-Mental State Examination (MMSE) scores greater than 24. Neuropsychological testing was used to determine clinical status using operational criteria for MCI as defined previously [21–24]. MCI designation was based on objective criteria of at least two performances within a cognitive domain falling >1 SD below published normative values.

Age-sex matched controls with normal cognition (NC) were selected to match the NDD cohort. One hundred and nine subjects with a Washington University Clinical Dementia Rating of zero were selected from the Knight’s Alzheimer’s Disease Research Center database, and 11 from the INSPECDS database of healthy subjects with MMSE ≥29. Recordings were excluded if two nights with at least 4 h of sleep time were unavailable, the between-nights difference in sleep time exceeded 1.5 h or if more than 10% of the recording time was rejected due to artifact.

### Data acquisition and reduction

**Fig.1:**
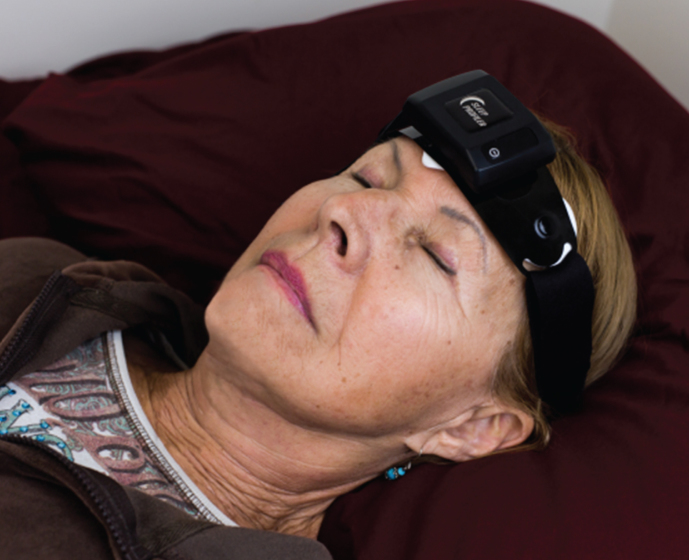
Photo of subject wearing the Sleep Profiler.

The multi-night recordings were acquired using the Sleep Profiler^TM^ (Advanced Brain Monitoring, Carlsbad, CA, US), a battery powered device affixed to the forehead. The device acquired electroencephalography (EEG), electrooculography, and electromyography from three frontopolar EEG signals AF7-AF8, AF7-Fpz, and AF8-Fpz (Fig. 1). Photoplethesmography obtained from the forehead was used to calculate the pulse rate. Snoring sounds were acquired with an acoustic microphone. Head movement and head position were derived from a triaxial accelerometer after signal conversion to 360° angles.

For the in-home studies, patients were instructed to wipe their forehead thoroughly with an alcohol wipe prior to affixing the device to obtain acceptable skin-sensor impedances. Voice messages alerted patients when the impedances were too high at the beginning of the night. Patients replaced the forehead sensors prior to Night 2.

Sleep Profiler Medical and History Questionnaires were acquired from participants at the time of their study; one NDD patient had missing data. The questionnaire was used to acquire demographic and anthropomorphic data, prior diagnoses of medical conditions, and use of medications. The questionnaire included a battery of screening questionnaires, including the Epworth Sleepiness Scale (ESS), Insomnia Severity Index (ISI), Patient Health Questionnaire Depression Test (PHQ9), and General Anxiety Disorder (GAD7). Eighteen of the healthy subjects included in the control cohort were previously diagnosed with OSA and 16 were treated with CPAP while undergoing their overnight EEG study. Of the 13 NDD patients who reported an OSA diagnosis, eight were being treated with CPAP prior to the study, two were untreated, and in three cases the treatment status could not be determined.

Once the studies were complete, the records and questionnaire responses were uploaded to the Sleep Profiler portal. Automated sleep staging was applied to sleep markers extracted from each 30 s epoch for the three frontopolar EEG channels. After rejection of periods contaminated with artifact a 0.75 Hz high pass filter was first applied, next a band stop filter was used to remove sweat artifact, and finally the signals band pass filtered at 16 Hz to obtain power values for delta, theta, alpha, sigma, beta, and EMG. Patterns in the AF7-Fpz and AF8-Fpz signals that characterized and distinguished slow rolling eye movements from phasic rapid eye movements were recognized by computing Pearson correlations of the infinite impulse response filtered outputs. The distinction between elevated delta power resulting from phasic REM versus slow wave sleep was made using the difference in delta power before and after ocular decontamination. The power spectra values were also used to detect sleep spindles (i.e., brief bursts in both alpha and sigma power), cortical arousals (i.e., elevated alpha for at least 3 s), and micro arousal events (i.e., combination of elevated alpha and/or EMG for at least 3 s). A description of the Sleep Profiler device, signal transformation, and automated staging rules, as well as the accuracy and reliability of the sleep metrics as compared to laboratory PSG were previously described [25–27]. For each 30 s epoch, the power spectra values were averaged, and the number of arousals, spindles, movements, snoring, position, and other patterns tallied. These data in combination with ratios of the mean power spectra were used to assign sleep stages using discriminant function analyses. One rater visually inspected the frontopolar EEG signal waveforms along with the presentations of the alpha, sigma, beta, and EMG power to confirm the accuracy of the auto-staging.

To evaluate nocturnal mobility (i.e., position changes), the head position at the start of each 30 s epoch was assigned. The file containing the head position across all epochs was edited to establish a modified recording time that excluded epochs at the start of the study when the subject was clearly settling into a sleeping position, and at the end of the study when the subject was awake and started moving to complete the study. Between these two time points, no effort was made to exclude head position changes which might bias the assessment of nocturnal mobility. Transitions from lateral left to lateral right commonly resulted in two position changes, i.e., lateral left to supine, and supine to lateral right. The transition from prone left to prone right was considered a head position change even though the torso likely did not change position. Head position changes per hour were derived from the total number of head position changes divided by the hours of modified recording time.

### Data analysis

The two nights of data, when available, were combined using a weighted average (e.g., Sleep efficiency = sleep time for nights 1 plus 2 divided by recording time for nights 1 plus 2). Independent *t*-test with the assumption of equal variance were used for comparisons of continuous variables. Pearson Chi-square analyses were used for comparisons of categorical data with at least 5 values in all conditions. Fisher exact probability tests were used for comparisons of categorical data with at least one condition total <5. Two-tailed probability tests were applied to all statistical analyses. Multivariable logistic regression was used to assess the strength of the relationship between NDD and the independent variables supine sleep >2 h/night, percent time supine, snoring, OSA, age, and sex.

Standard severity scoring rules were applied to the ISI, PHQ9, and GAD7. For the ESS, mild ranged from 11 to 13, moderate 14 to 16, and severe >16.

## RESULTS

### Demographic, comorbidity, and symptomatology

There were no significant differences between NC and NDD group based on age, sex, BMI, or presence of diabetes or heart disease (Table 1). NC subjects had significantly greater hypertension diagnoses and antihypertensive medication prescription than the NDD group. Conversely, NDD patients had significantly more frequent OSA diagnosis than the NC group. Overall, the NC had higher ESS scores compared to the NDD, a result of slightly elevated mean scores for those with ESS <10 (NC: 5.1 ±2.6 versus NDD: 3.4±2.5, *p* = 0.001) (Table 2). The NDD group reported higher ISI scores and a greater percentage reported abnormal ISI scores (*p* = 0.01) compared to NC. Although the NDD group reported significantly higher PHQ7 scores, there were no significant proportional differences between the two groups. The use of antidepressant (NDD = 30% versus NC = 21%, *p* = 0.22), anxiolytic, and prescription hypnotic medications (less than 7% and 2.4%, respectively) were similar across groups. For the NDD group, 16 were taking acetylcholinesterase inhibitor (AChEl), 11 were taking serotonin reuptake inhibitor (SSRI), while four received antihypertensive therapy, including two on alpha blockers and two on beta blockers. Sub-classed medication information was not available for the NC group.

**Table 1:** Descriptive statistics

	NC	NDD	*P*
Demographics			
Subjects; n	120	45	–
Age; y	71.9±6.8	70.9±7.9	0.397
Women; % (*n*)	39.2 (47)	33.3 (15)	0.470
Body mass index; kg/m^2^	26.9±5.5	25.6±4.0	0.129
Comorbidities			
Hypertension; % (*n*)	46.7 (56)	22.7 (10)	0.006
Diabetes; % (*n*)	6.7 (8)	4.5 (2)	0.731
Heart disease; % (*n*)	15.0 (18)	13.6 (6)	0.823
OSA; % (*n*)	15.0 (18)	29.5 (13)	0.035
Insomnia; % (*n*)	5.8 (7)	9.1 (4)	0.488
Depression; % (*n*)	18.3 (22)	25.0 (11)	0.345
Restless Legs; % (*n*)	6.7 (8)	2.3 (1)	0.282

**Table 2:** Self-reported symptomatology by cognition state

	Mean + SD	*p*	Normal	Mild	Moderate	Severe
Epworth Sleepiness Scale (ESS)						
NC	6.1±3.7	0.034	85.9%	10.8%	2.5%	0.8%
NDD	4.7±4.7		88.7%	2.3%	4.5%	4.5%
Insomnia Severity Index (ISI)						
NC	4.6±3.8	0.011	79.0%	17.6%	3.4%	0.0%
NDD	6.5±5.8		59.1%	29.5%	9.1%	2.3%
PHQ9 Depression score						
NC	2.0±2.2	0.032	84.2%	15.0%	0.8%	0.0%
NDD	3.0±3.9		79.5%	13.6%	4.6%	2.3%
GAD7 Anxiety score						
NC	1.4±2.2	0.159	91.7%	6.6%	1.7%	0.0%
NDD	2.0±3.3		81.8%	15.9%	0.0%	2.3%

### Sleep position metrics

The NDD cohort spent significantly more sleep time with the head in the supine position based on the percent time supine (*p* < 0.0001) and the proportion of those who sleep supine >2 h/night (*p* < 0.0001, odds ratio 3.7, 95% CI 1.8 to 7.7) (Table 3). Significant differences were observed in supine time >2 h/night when the NC no OSA sub-group was compared to the NDD no OSA sub-group (*p* < 0.001). No difference in frequency of supine sleep >2 h/night was observed across the MCI, AD, or PD/DLB/other dementia sub-groups (all *p* > 0.60). A multivariable logistic analysis confirmed that both supine sleep >2 h/night (*p* = 0.01) and the percentage of time supine (*p* = 0.001) were associated with NDD, but not age, sex, obstructive sleep apnea, or snoring.

**Table 3:** Sleep architectural features by cognition state and OSA

Measure	NC group			NDD group			Group *p*
	No OSA	OSA	*p*	No OSA	OSA	*p*
Sleep time; h	6.2±1.0	6.4±0.8	0.35	6.2±1.8	6.5±0.7	0.56	0.75
Sleep efficiency; %	79.6±8.9	83.1±6.2	0.11	78.5±13.2	82.6±9.4	0.32	0.79
Time supine; %	30.3±27.9	31.9±28.8	0.83	51.7±30.7	56.0±30.3	0.67	<0.001
% >2 h supine sleep	37.3	38.9	0.89	71.9	61.5	0.72	<0.001
Head position changes; h	2.3±1.1	1.9±1.2	0.32	2.1±1.8	2.0±0.7	0.99	0.47

The number of head position changes/h were similar between groups and OSA sub-groups, providing evidence that the differences in supine time were likely not driven by decreased mobility in the NDD group. However, the percent time supine and the number of position changes/h were correlated in both the NDD and NC groups (NDD: *r* = 0.34, *p* = 0.02 versus NC: *r* = 0.33, *p* < 0.001, respectively).

## DISCUSSION

This study reports the ambulatory sleep patterns and predominant habitual sleep positions of a clinically well characterized cognitive cohort enrolled in longitudinal studies conducted across multiple institutions. This was our first report into the investigation of sleep biomarkers that might be detectable across a range of severities in patients with etiologies associated with increased concentrations of CNS neurotoxins and metabolites [28].

To our knowledge, this study is the first to show a relationship between time spent in the supine sleep position and dementia. We demonstrated that supine sleep >2 h/night was significantly more frequent in patients with NDD, and supine sleep >2 h/night was independently associated with NDD when adjusting for sex, age, OSA diagnosis, or snoring. One possible explanation for this finding is that gravity affects the movement and distribution of blood out of the brain, and therefore head position during sleep could affect the efficiency of protein clearance from the brain [14–18]. Lee et al. observed glymphatic transport was more efficient in the lateral position as compared to the supine or prone positions in sleeping rats [19]. The interaction between aging and sleep could further affect the efficiency of this glymphatic clearance. First, breathing rates during sleep increase with age, likely as a result of decreased lung efficiency. Shallower breaths would reduce the magnitude of positive intrathoracic pressure and thus lower mean intracranial pressure. Second, the magnitude of penetrating arterial pulsatility in the brain decreases with age [29]. These factors could result in less efficient clearance associated with each breath (i.e., clearance cycle). The natural age-related decrease in sleep duration [30] would further contribute by reducing the number of clearance cycles per night. We purposely associated NDD with prolonged supine sleep duration (i.e., >2 h/night) to avoid misinterpretations resulting from use of percentage of supine sleep conjoined to short duration studies. We acknowledge that reduced mobility in NDD subjects could account for the increased duration spent in the supine position, however we did not observe differences between the groups in the number of head position changes per hour. There was a weak association between nocturnal supine head position time and position changes/h in both NDD and NC groups, likely due to the number of position changes that are associated with transitioning between lateral sleep positions.

One limitation of this study is that a prior diagnosis of OSA was used for stratification purposes rather than exclusion criteria, due to the limited sample size and retrospective case-matching design. Although a significantly greater percentage of NDD had been diagnosed with OSA, indications of undiagnosed OSA in the NC group included twice the prevalence of hypertension and significantly greater daytime somnolence as compared to the NDD group. Given the high prevalence of OSA in the elderly, it was not surprising that at least 10% of non-OSA cognitively normal and NDD patients’ records included evidence of sleep disordered breathing based on the Sleep Profiler signals. This observation was made during the focused review based on the overlapping timing of crescendo or brief snores with autonomic activations, head movements, and/or cortical or micro-arousals.

Our findings may have interesting implications for the field of neurodegeneration, in light of data which has suggested a link between supine sleep position and reduced glymphatic clearance in sleeping rats. In humans, it is currently not readily feasible to directly measure position-related changes in the glymphatic clearance of soluble proteins such as Aβ. We analyzed NDD patients with diagnoses of MCI, AD, PD, and DLB, disorders associated with the accumulation of Aβ, alpha-synuclein, and tau proteins in the brain [31]. We found a significant association between supine sleep >2 h/night and patients with established diagnoses of these neurodegenerative diseases. This cross-sectional data certainly does not prove causation of pathological protein accumulation due to a greater duration of supine sleep, since temporal association cannot be evaluated. Given the ease and relatively low cost by which the supine head position could be measured and avoided, our findings suggest that home sleep architectural and positional monitoring should be explored in future longitudinal cohort studies of NDD patients who undergo routine serial clinical assessment and imaging. The frequency and duration of the head in the supine position during sleep could be monitored in these NDD patients and correlated with clinical, imaging, and CSF neurodegenerative markers. Future studies could also include NDD, OSA, and control groups undergoing an intervention involving sleep positional avoidance feedback training to restrict supine sleep duration and measure these same longitudinal markers for neurodegenerative disease status and progression.

### Conclusions

We found that home supine sleep position was independently associated with neurodegenerative disease. Our findings suggest the intriguing possibility that head position during sleep could influence the clearance of neurotoxic proteins from the brain. Future larger prospective longitudinal observational cohort studies of sleep position and architecture in neurodegenerative disease patients will be necessary to determine the relevance and directionality of these findings toward neurocognitive performance and clinical, imaging, and CSF markers of neurodegenerative disease burden.

## References

[1] Ju YE , Lucey BP , Holtzman DM (2014) Sleep and Alzheimer disease pathology–a bidirectional relationship. Nat Rev Neurol 10, 115–119. 24366271 10.1038/nrneurol.2013.269 PMC3979317

[2] Hung CM , Li YC , Chen HJ , Lu K , Liang CL , Liliang PC , Tsai YD , Wang KW (2018) Risk of dementia in patients with primary insomnia: A nationwide population-based case-control study. BMC Psychiatry 18, 38. 29415688 10.1186/s12888-018-1623-0 PMC5804010

[3] Lutsey PL , Misialek JR , Mosley TH , Gottesman RF , Punjabi NM , Sharar E , MacLehose R , Ogilvie RP , Knopman D , Alonso A (2018) Sleep characteristics and risk of dementia and Alzheimer’s disease: The Atherosclerosis risk in communities study. Alzheimers Dement 14, 157–166. 28738188 10.1016/j.jalz.2017.06.2269 PMC5776061

[4] Tarasoff-Conway JM , Carare RO , Osorio RS , Glodzik L , Butler T , Fieremans E , Axel L , Rusinek H , Nicholson C , Zlokovic BV , Frangione B , Blennow K , Menard J , Zetterberg H , Wisniewski T , de Leon MJ (2015) Clearance systems in the brain-implications for Alzheimer disease. Nat Rev Neurol 11, 457–470. 26195256 10.1038/nrneurol.2015.119 PMC4694579

[5] Plog BA , Nedergaard M (2018) The glymphatic system in central nervous system health and disease: Past, present and future. Annu Res Pathol 13, 379–394. 10.1146/annurev-pathol-051217-111018 PMC5803388 29195051

[6] Iliff JJ , Chen MJ , Plog BA , Zeppenfeld DM , Soltero M , Yang L , Singh I , Deane R , Nedergaard M (2014) Impairment of glymphatic pathway function promotes tau pathology after traumatic brain injury. J Neurosci 34, 16180–16193. 25471560 10.1523/JNEUROSCI.3020-14.2014 PMC4252540

[7] Shokri-Kojori E , Wang GJ , Wiers CE , Demiral SB , Guo M , Kim SW , Lindgren E , Ramirez V , Zehra A , Freeman C , Miller G , Manza P , Srivastava T , De Santi S , Tomasi D , Benveniste H , Volkow ND (2018) β-amyloid accumulation in the human brain after one night of sleep deprivation. Proc Natl Acad Sci U S A 115, 4483–4488. 29632177 10.1073/pnas.1721694115 PMC5924922

[8] Lucey BP , Hicks TJ , McLeland JS , Toedebusch CD , Boyd J , Elbert DL , Patterson BW , Baty J , Morris JC , Ovod V , Mawuenyega KG , Bateman RJ (2018) Effect of sleep on overnight cerebrospinal fluid amyloid β kinetics. Ann Neurol 83, 197–204. 29220873 10.1002/ana.25117 PMC5876097

[9] Huang Y , Potter R , Sigurdson W , Kasten T , Connors R , Morris JC , Benzinger T , Mintun M , Ashwood T , Ferm M , Budd SL , Bateman RJ (2012) β-amyloid dynamics in human plasma. Arch Neurol 69, 1591–1597. 23229043 10.1001/archneurol.2012.18107 PMC3808092

[10] Varga AW , Wohlleber ME , Giménez S , Romero S , Alonso JF , Ducca EL , Kam K , Lewis C , Tanzi EB , Tweardy S , Kishi A , Parekh A , Fischer E , Gumb T , Alcolea D , Fortea J , Lleo A , Blennow K , Zetterberg H , Mosconi L , Glodzik L , Pirraglia E , Burschtin OE , de Leon MJ , Rapoport DM , Lu SE , Ayappa I , Osorio RS (2016) Reduced slow-wave sleep is associated with high cerebrospinal fluid Aβ42 levels in cognitively normal elderly. Sleep 39, 2041–2048. 27568802 10.5665/sleep.6240 PMC5070758

[11] Sharma RA , Varga AW , Bubu OM , Pirraglia E , Kam K , Parekh A , Wohlleber M , Miller MD , Andrade A , Lewis C , Tweardy S. Buj M , Yau PL , Sadda R , Mosconi L , Li Y , Butler T , Glodzik L , Fieremans E , Babb JS , Blennow K , Zetterberg H , Lu SE , Badia SG , Romero S , Rosenzweig I , Gosselin N , Jean-Louis G , Rapoport DM , de Leon MJ , Ayappa I , Osorio RS (2018) Obstructive sleep apnea severity affects amyloid burden in cognitive normal elderly. A longitudinal study. Am J Respir Crit Care Med 197, 933–943. 29125327 10.1164/rccm.201704-0704OC PMC6020410

[12] Ooms S , Overeem S , Besse K , Rikkert MO , Verbeek M , Claassen JA (2014) Effect of 1 night of total sleep deprivation on cerebrospinal fluid β-amyloid 42 in healthy middle-aged men: A randomized clinical trial. JAMA Neurol 71, 971–977. 24887018 10.1001/jamaneurol.2014.1173

[13] Ju YE , Finn MB , Sutphen CL , Herries EM , Jerome GM , Ladenson JH , Crimmins DL , Fagan AM , Holtzman DM (2016) Obstructive sleep apnea decreases central nervous system-deprived proteins in the cerebrospinal fluid. Ann Neurol 80, 154–159. 27129429 10.1002/ana.24672 PMC5120585

[14] Torbey MT , Geocadin RG , Razumovsky AY , Rigamonti D , Williams MA (2004) Utility of CSF pressure monitoring to identify idiopathic intracranial hypertension without papilledema in patients with chronic headache. Cephalalgia 24, 495–502. 15154860 10.1111/j.1468-2982.2004.00688.x

[15] Gooding CA , Stimac GK (1984) Jugular vein obstruction caused by turning of the head. AJR Am J Roentgenol 142, 403–406. 6421115 10.2214/ajr.142.2.403

[16] Zaniewski M , Simka M (2012) Biophysics of venous return from the brain from the perspective of the pathophysiology of chronic cerebrospinal venous insufficiency. Rev Recent Clin Trials 7, 88–92. 22338621 10.2174/157488712800100288

[17] Højlund J , Sandmand M , Sonne M , Mantoni T , Jorgensen HL , Belhage B , Lieshout JJ , Pott FC (2012) Effect of head rotation on cerebral blood velocity in the prone position. Anesthesiol Res Pract 2012, 647258. 22988456 10.1155/2012/647258 PMC3440850

[18] Barami K (2016) Cerebral venous overdrainage: An under-recognized complication of cerebrospinal fluid diversion. Neurosurg Focus 41, E9. 10.3171/2016.6.FOCUS16172 27581321

[19] Lee H , Xie L , Yu M , Kang H , Feng T , Deane R , Logan J , Nedergaard M , Benveniste H (2015) The effect of body posture on brain glymphatic transport. J Neurosci 35, 11034–11044. 26245965 10.1523/JNEUROSCI.1625-15.2015 PMC4524974

[20] McKeith IG , Boeve BF , Dickson DW , Halliday G , Taylor JP , Weintraub D , Aarsland D , Galvin J , Attems J , Ballard CG , Bayston A , Beach TG , Blanc F , Bohnen N , Bonanni L , Bras J , Brundin P , Burn D , Chen-Plotkin A , Duda JE , El-Agnaf O , Feldman H , Ferman TJ , Ffytche D , Fujishiro H , Galasko D , Goldman JG , Gomperts SN , Graff-Radford NR , Honig LS , Iranzo A , Kantarci K , Kaufer D , Kukull W , Lee VMY , Leverenz JB , Lewis S , Lippa C , Lunde A , Masellis M , Masliah E , McLean P , Mollenhauer B , Montine TJ , Moreno E , Mori E , Murray M , O’Brien JT , Orimo S , Postuma RB , Ramaswamy S , Ross OA , Salmon DP , Singleton A , Taylor A , Thomas A , Tiraboschi P , Toledo JB , Trojanowski JQ , Tsuang D , Walker Z , Yamada M , Kosaka K (2017) Diagnosis and management of dementia with Lewy bodies; Fourth consensus report of the DLB Consortium. Neurology 89, 88–100. 28592453 10.1212/WNL.0000000000004058 PMC5496518

[21] Bondi MW , Edmonds EC , Jak AJ , Clark LR , Delano-Wood L , McDonald CR , Nation DA , Libon DJ , Au R , Galasko D , Salmon DP (2014) Neuropsychological criteria for mild cognitive impairment improves diagnostic precision, biomarker associations, and progression rates. J Alzheimers Dis 42, 275–289. 24844687 10.3233/JAD-140276 PMC4133291

[22] Bondi MW , Jak AJ , Delano-Wood L , Jacobson MW , Delis DC , Salmon DP (2008) Neuropsychological contributions to early identification of Alzheimer’s disease. Neuropsychol Rev 18, 73–90. 18347989 10.1007/s11065-008-9054-1 PMC2882236

[23] Jak AJ , Bondi MW , Delano-Wood L , Wierenga C , Corey-Bloom J , Salmon DP , Delis DC (2009) Quantification of five neuropsychological approaches to defining mild cognitive impairment. Am J Geriatr Psychiatry 17, 368–375. 19390294 10.1097/JGP.0b013e31819431d5 PMC2743175

[24] Stricker NH , Salat DH , Foley JM , Zink TA , Kellison IL , McFarland CP , Grade LJ , McGlinchey RE , Milberg WP , Leritz EC (2013) Decreased white matter integrity in neuropsychologically defined mild cognitive impairment is independent of cortical thinning. J Int Neuropsychol Soc 19, 925–937. 23809097 10.1017/S1355617713000660 PMC4356249

[25] Levendowski DJ , Ferini-Strambi L , Gamaldo C , Cetel M , Rosenberg R , Westbrook P (2017) The accuracy, night-to-night variability and stability of frontopolar sleep electroencephalography biomarkers. J Clin Sleep Med 13, 791–803. 28454598 10.5664/jcsm.6618 PMC5443740

[26] Stepnowsky C , Levendowski D , Popovic D , Ayappa I , Rapoport DM (2013) Scoring accuracy of automated sleep staging from a bipolar electroocular recording compared to manual scoring by multiple raters. Sleep Med 14, 1199–207. 24047533 10.1016/j.sleep.2013.04.022

[27] Finan PH , Richards JM , Gamaldo CE , Han D , Leoutsakos JM , Salas R , Irwin MR , Smith MT (2016) Validation of a wireless, self-application, ambulatory electroencephalographic sleep monitoring device in healthy volunteers. J Clin Sleep Med 12, 1443–1451. 27707438 10.5664/jcsm.6262 PMC5078698

[28] Vergallo A , Bun RS , Toschi N , Baldacci F , Zetterberg H , Blennow K , Cavedo E , Lamari F , Habert MO , Dubois B , Floris R , Garaci F , Lista S , Hampel H , INSIGHT-preAD study group, Alzheimer Precisiion Medicine Initiative (APMI) (2018) Association of cerebrospinal fluid *α*-synuclein with total phospho-tau_181_ protein concentrations and brain amyloid load in cognitively normal subject memory complainers stratified by Alzheimer’s disease biomarkers. Alzheimers Dement 14, 1623–1631. 30055132 10.1016/j.jalz.2018.06.3053

[29] Kress BT , Iliff JJ , Xia M , Wang M , Wei HS , Zeppenfeld D , Xie L , Kang H , Xu Q , Liew JA , Plog BA , Ding F , Deane R , Nedergaard M (2014) Impairment of paravascular clearance pathways in the aging brain. Ann Neurol 76, 845–861. 25204284 10.1002/ana.24271 PMC4245362

[30] Åkerstedt T , Discacciati A , Miley-Åkerstedt A , Westerlund H (2018) Aging and the change in fatigue and sleep - a longitudinal study across 8 years in three age groups. Front Psychol 9, 234. 29568279 10.3389/fpsyg.2018.00234 PMC5852064

[31] Lowe VJ , Wiste HJ , Senjem ML , Weigand SD , Theneau TM , Boeve BF , Josephs KA , Fang P , Pandey MK , Murray ME , Kantarci K , Jones DT , Vemuri P , Graff-Radford J , Schwartz CG , Machulda MM , Mielke MM , Roberts RO , Knopman DS , Petersen RC , Jack CR Jr (2018) Widespread brain tau and its association with ageing, Braak stage and Alzheimer’s dementia. Brain 141, 271–287. 29228201 10.1093/brain/awx320 PMC5837250

